# Intracellular Trafficking of HBV Particles

**DOI:** 10.3390/cells9092023

**Published:** 2020-09-02

**Authors:** Bingfu Jiang, Eberhard Hildt

**Affiliations:** 1 Department of Virology, Paul-Ehrlich-Institut, D-63225 Langen, Germany; jiang.bingfu@outlook.com; 2 German Center for Infection Research (DZIF), TTU Hepatitis, Marburg-Gießen-Langen, D63225 Langen, Germany

**Keywords:** hepatitis B virus, trafficking, entry, morphogenesis, release

## Abstract

The human hepatitis B virus (HBV), that is causative for more than 240 million cases of chronic liver inflammation (hepatitis), is an enveloped virus with a partially double-stranded DNA genome. After virion uptake by receptor-mediated endocytosis, the viral nucleocapsid is transported towards the nuclear pore complex. In the nuclear basket, the nucleocapsid disassembles. The viral genome that is covalently linked to the viral polymerase, which harbors a bipartite NLS, is imported into the nucleus. Here, the partially double-stranded DNA genome is converted in a minichromosome-like structure, the covalently closed circular DNA (cccDNA). The DNA virus HBV replicates via a pregenomic RNA (pgRNA)-intermediate that is reverse transcribed into DNA. HBV-infected cells release apart from the infectious viral parrticle two forms of non-infectious subviral particles (spheres and filaments), which are assembled by the surface proteins but lack any capsid and nucleic acid. In addition, naked capsids are released by HBV replicating cells. Infectious viral particles and filaments are released via multivesicular bodies; spheres are secreted by the classic constitutive secretory pathway. The release of naked capsids is still not fully understood, autophagosomal processes are discussed. This review describes intracellular trafficking pathways involved in virus entry, morphogenesis and release of (sub)viral particles.

## 1. Introduction

**Figure 1:**
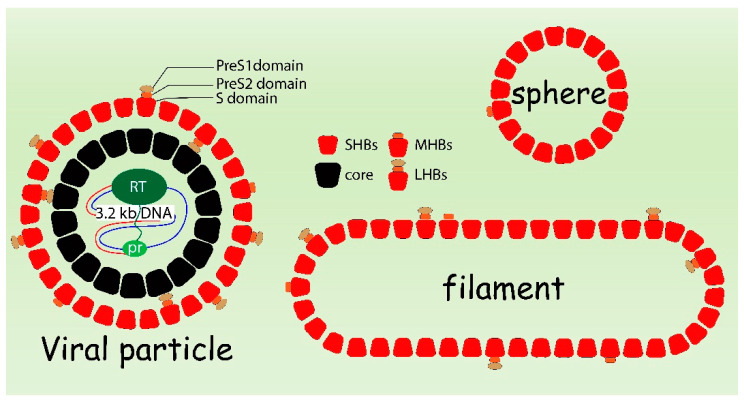
Schematic presentation of HBV viral particle, subviral filament and sphere.

The human hepatitis B virus (HBV) belongs to the family of Hepadnaviridae, a group of small hepatotropic DNA viruses. HBV is an enveloped virus. The three viral surface proteins are LHB (large hepatitis B virus surface protein), MHB (middle hepatitis B virus surface protein) and SHB (small hepatitis B virus surface protein) that are integral membrane proteins embedded into the membrane. The membrane surrounds the nucleocapsid. The nucleocapsid is assembled by the core protein (HBc) and harbors the viral DNA genome. The viral genome is a partially double-stranded DNA with a size of about 3.2 kb. This very compact genome encompasses four overlapping open reading frames (ORFs) encoding the viral polymerase (P), the capsid forming core protein (HBc) and its secretory variant HBeAg, the regulatory protein HBx, and the three envelope proteins—LHBs, MHBs and SHBs [1,2,3]. In addition to being part of the infectious viral particle, the surface proteins can also form non-infectious subviral particles (SVPs) that lack capsid and genome. The SVPs exist as spheres with a diameter of 22 nm and filaments with a variable length between 80 and 400 nm (Figure 1).

The HBV envelope proteins, that are also termed HBV surface antigens (HBsAg), are encoded by a single open reading frame that is divided by three in frame standing start codons in the PreS1, the PreS2 and the S region. The three surface proteins share the S-domain with 226 amino acids (aa), which forms SHBs. MHBs contains an additional PreS2 domain with 55 aa, while LHBs encompasses the PreS2 domain and the PreS1 domain with a length of 108, 118 or 119 aa depending on the genotype. The HBV surface proteins are integral membrane proteins anchored by the S-domain in the membrane. SHBs is inserted by various transmembrane (TM) regions in the ER membrane. The first ER transmembrane region (TM1) encompasses aa 8–22 [4], which is followed by a cytosolic loop aa 23–79. The second transmembrane domain (TM2) (aa 80–98) directs the luminal exposure of aa 99–169 containing the major conformational epitope of HBsAg (a-determinant aa 124–147) and a N-glycosylation site [5]. The detailed structure of the following C-terminal sequence has not yet been studied experimentally, but it has been considered as one or two transmembrane regions (TM3/4) [5,6]. MHBs shows no difference in the topology of its S-domain as compared to SHBs. The PreS2 domain of MHBs directs in the ER lumen and is N-glycosylated at Asn-4 [7]. The N-terminus of LHBs is subjected to myristoylation. Integrity of the myristoylation site is crucial for the infectivity of HBV [8,9]. With respect to the PreS1PreS2 domain, LHBs is characterized by a dual topology: The TM1 of nascent LHBs does not function as a co-translational translocation signal sequence, leading to the cytosolic exposure of the PreS1PreS2 domain and S-domain up to aa 79. However, due to a posttranslational translocation, about 50% of TM1 are integrated in the ER membrane. In this case, the PreS1PreS2 domain and the first 7 aa of the S-domain face the lumen of the ER, [10,11,12]. The PreS-domain directing in the ER lumen (e-PreS) is exposed on the viral surface and mediates the virus–cell interaction [13], while the one facing the cytosol (i-PreS) has been postulated to interact with the nucleocapsid during viral envelopment [14]. Moreover, the PreS2 domain facing the cytoplasm acts as a regulatory protein that binds and activates classic isoforms of PKC that leads to an activation of c-Raf-dependent signaling cascades. For more information, readers are referred to pertinent reports [15,16].

The HBV core protein (HBc, capsid protein) contains genotype-dependent 183 or 185 aa. The primary structure of HBc can be divided into an N-terminal domain (NTD, also designated as assembly domain) and a C-terminal domain (CTD), which are connected by a linker region. The NTD consists of the first N-terminal 149 or 151 aa (depending on the genotype), which are sufficient for the self-assembly of viral capsids [17,18,19]. The structure of the core polypeptide is characterized by a highly α-helical fraction. Two α-helices (α3, α4) connected by a loop constitute the central domain of a monomer. It is assumed to be linked by a disulfide bridge, the stem constitutes the dimer interface and exposes the spikes on the outer surface of the icosahedral capsid [20,21]. However, there is evidence that fully oxidized dimers assemble slower into capsids as compared to the reduced dimers. Apart from the velocity of capsid formation, the stability of the formed capsid is affected by the oxidation of the dimer. It is assumed that capsid destabilization by the Cys61-Cys61 disulfide bond formation fulfills a regulatory function with respect to the capsid disassembly [19].

The formation of HBc dimers is a prerequisite for capsid assembly [22,23]. In general, two different capsids characterized by the triangulation numbers T = 3 and T = 4 can be assembled by the HBc dimers. The capsid in T = 3 symmetry consists of 90 HBc dimers with a diameter of 30 nm, while the T = 4 symmetry particle is composed of the 120 HBc dimers, having a diameter of 34 nm [24,25]. The two classes of capsids can be separated on sucrose gradients and do not interconvert upon protracted storage [24]. Though both types of capsids can be observed in HBc-producing systems, the majority is the T = 4 icosahedral symmetry preferentially incorporated into virions [26,27]. However, there is evidence that virions are formed harboring capsids with T = 3 symmetry [27]. While NTD is essential for the capsid assembly in vitro, the CTD is not relevant for capsid assembly [23,28]. In the rabbit reticulocyte lysate system and in HuH7 cells the CTD is required for assembly [29,30]. Being rich in arginine, CTD possesses non-specific nucleic acid-binding activity, and is essential for pgRNA packaging and reverse transcription [28,31,32]. During pgRNA packaging and reverse transcription, the CTD undergoes dynamic phosphorylation and dephosphorylation to regulate HBc functions [33,34]. In addition, nuclear localization signals (NLS) and nuclear export signals (NES) have been described in the CTD, which enables the cytoplasmic shuttling of capsids [35,36]. For recombinant expression and capsid assembly, the HBc linker is generally included together with NTD [37]. The linker seems to exert no function in capsid assembly as NTDs lacking the linker are able to assemble into capsids, though lack of the linker was shown to affect the ratio of the T = 3 and T = 4 symmetry capsid [24,38,39]. A recent study reported that the linker is relevant to HBV replication (e.g., pgRNA packaging, viral DNA synthesis, and the ratio of complete to empty virions) [40]. Recent reports provide an excellent overview about the structure of the different types of core dimers and capsids [41,42].

## 2. Intracellular Cytoplasmic Transport after Viral Uptake

A major progress for the understanding of HBV life cycle was the identification of sodium taurocholate cotransporting polypeptide (NTCP) as entry relevant factor or HBV [43]. The identification of NTCP as an entry relevant factor was associated with the observation that myristoylated peptides covering the N-terminus of the PreS1 domain efficiently block HBV infection [44,45].

**Figure 2:**
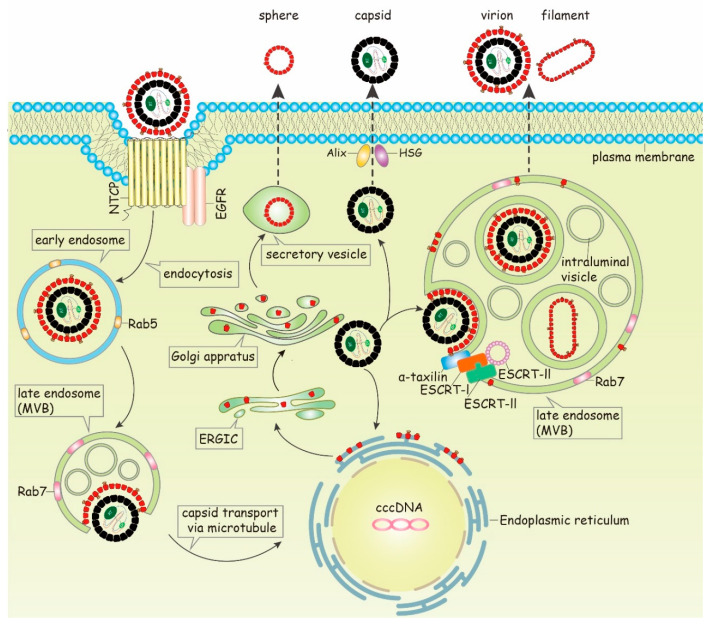
Model of HBV intracellular trafficking. After binding to the receptor complexes (NTCP = Sodium taurocholate cotransporting polypeptide), the virions are transported to Rab5-containing early endosomes followed by the Rab7-containing late endosomes. After their release into cytoplasm, the nucleocapsids are delivered to the nuclear pore complex by a directed transport involving microtubules. In the nuclear pore complex the nucleocapsid disassembles and the viral DNA genome is imported into the nucleus and converted to cccDNA (covalently closed circular DNA). In the early phase of the infection process, de novo synthesized nucleocapsids are transported back to the nuclear pore complex to augment the cccDNA pool in the nucleus. Later in the infection process, the surface proteins are synthesized at the rough ER (endoplasmatic reticulum) and transported through the ERGIC (ER-Golgi-intermediate-complex). Spheres are secreted by the constitutive secretory pathway. After envelopment of the nucleocapsid, the release of virions as well as of filaments is ESCRT/MVB-dependent and mediated by α-taxilin. In addition, non-enveloped assembled (nucleo)capsids are secreted as naked capsids involving Alix and HSG. (MVB = multivesicular bodies; *ESCRT =* endosomal sorting complexes required for transport; EGFR = epidermal growth factor receptor)

Years before NTCP was identified as entry factor, there was already evidence that HBV enters the cell by receptor-mediated endocytosis [46] (Figure 2). This step depends on cellular factors, such as caveolin-1 and dynamin-2, as significant reduction of HBV transcripts and antigens was observed in HepaRG cells expressing mutants of caveolin-1 and dynamin-2 [47]. A recent report described that HBV entry is independent on lipid raft-/caveolin-mediated endocytosis, but that clathrin-mediated endocytosis mediates the NTCP-dependent entry of HBV [48]. Knockdown of clathrin heavy chain (CHC), dynamin-2 and clathrin adaptor protein AP-2 reduced the susceptibility to HBV [49].

Furthermore, endosome-associated cellular Rab GTPases Rab5A and Rab7A have been demonstrated to be involved in transporting of HBV viral particles from early endosomes (rich of Rab5) to late endosomes (rich of Rab7) [50]. Silencing of either Rab5A or Rab7A leads to a significant inhibition of the early HBV infection, and a considerable number of virions were found in the late endosomes in a time-dependent manner. In contrast to this, silencing of Rab9 and Rab11, that are involved in the transport of the endocytotic vesicle to the recycling endosome or the trans Golgi complex, has no effect on the HBV entry. Although HBV viral particles are further transported to lysosome compartment, viral infectivity does not depend on the lysosomal activity [50]. A recent report described that the machinery for endocytosis of epidermal growth factor receptor (EGFR) coordinates the transport of incoming hepatitis B virus to the endosomal network [51,52]. This process involves EGF-R phosphorylation and the subsequent recruitment of adaptor molecules such as AP2A1 and Eps15. The EGFR-sorting machinery coordinates HBV transport in the endosomal network (early and late endosome and lysosome). In accordance to this, suppression of EGFR ubiquitination impairs HBV infection [52]. These observations imply that translocation of the virus to late endosomes is critical for a successful infection. However, how the viral particles deliver their nucleocapsids from endosomal compartments into the cytoplasm was not clearly elucidated in these studies.

Previous reports described the identification of a fusogenic motive in the PreS1 domain [53,54]. On the one hand, there are data that identify aa 9–24 (NPLGFFPDHQLDPAFG) of the PreS1 domain as a membrane destabilizing sequence that could be involved in the escape from the endosomal compartment [55]. There are reports that describe that a defined pattern of aa residues in the PreS1 domain is causative for the fusogenic activity [56]. In addition, the peptide encompassing aa 7–18 of the S-domain was discussed to be involved in the entry process by mediating hemifusion [57]. In accordance to this, data based on the DHBV system suggest that the first transmembrane region in the S-domain is involved in the fusion process [58].

**Figure 3:**
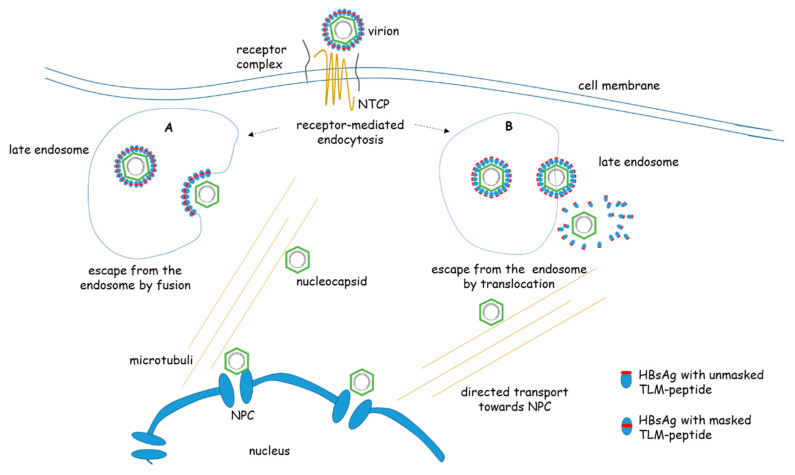
Entry and post-entry steps of HBV. HBV enters the cell by binding to a receptor/receptor-complex containing NTCP (Sodium taurocholate cotransporting polypeptide). The internalization occurs by receptor-mediated endocytosis. This cartoon shows two models that describe the escape from the late endosome: A) by membrane fusion leading to the release of the nucleocapsid into the cytoplasm. B) By a translocation process mediated by the TLM peptide. In the mature virus the TLM (red dot) is masked. Due to proteolytic processing of the surface protein in the endosomal compartment the TLM is unmasked and exposed to the surface of the viral particle. The surface-exposed TLM peptides mediate the translocation across the endosomal membrane into the cytoplasm. Here the processed surface proteins are stripped off. The cytoplasmic nucleocapsid is delivered by a directed transport involving microtubule to the nuclear pore complex (NPC). (NTCP = Sodium taurocholate cotransporting polypeptide, TLM = translocation motive)

A fundamental different mechanism was proposed based on the identification of a conserved membrane permeable peptide in the surface protein of HBV. A cell-permeable peptide was identified between the amino acids 41 and 52 of the PreS2 domain. The peptide has been designated as translocation motif (TLM). It has been described as a novel tool for the delivery of proteins and nucleic acids into tissues and cells [59,60,61,62,63,64,65]. The functionality of TLM depends on a defined pattern of hydrophilic and hydrophobic amino acids that form a labile amphipathic alpha helix [60,62,64,66]. Fusion of the TLM to HBc enables the formation of fully-assembled capsids (VLPs) that are decorated with the TLM-peptide. These VLPs translocate as complete particles across the plasma membrane and transverse the cytoplasm toward the nucleus [65]. A marker gene packaged into these capsids is efficiently expressed. Thus, these TLM-capsids could represent a tool to study intracellular trafficking of HBV-capsids in intact cells (Figure 3).

The presence of a TLM in the PreS-domain is conserved in all hepadnaviruses, suggesting a relevance for the viral life cycle. In the mature virus, the TLM is masked and not exposed on the viral surface. It was revealed that destruction of the TLM neither affects the binding nor the entry of these mutant viruses into the cells permissive for HBV infection. Further analyses revealed that these mutants are trapped in the endosomal compartment. Treatment of viral particles with endosomal lysates caused a proteolytic processing of the viral envelope proteins leading to a conformational change that unmasks the TLM. This processing enabled a receptor independent “infection” of non-permissive cells due to the TLM-dependent translocation of the processed virus. Based on these data, it was concluded that, after receptor mediated endocytosis, a proteolytic processing of the viral particle occurs, leading to the unmasking of the TLMs on the viral surface that enable the translocation across the endosomal membrane. In the cytoplasm the destabilized envelope is removed and the released nucleocapsid is transferred to the nuclear pore complex by a directed transport [67].

There are conflicting data about the relevance of the TLM for the HBV entry process. Based on HDV experiments and mutants harboring a mutant TLM, it was reported that the TLM is dispensable for HBV infectivity [68,69,70].

After the escape from the endosomal compartments (Figure 3), the nucleocapsids have been assumed to be delivered to the nucleus via microtubule-mediated transport [71], a mechanism exploited by many viruses, including retroviruses, herpesviruses, parvoviruses, and adenoviruses [72,73]. The intracellular localization of capsids showed a very close correspondence to the intracytoplasmic distribution of tubulin, a major protein of the microtubule network. Moreover, depolymerization of microtubules with nocodazole blocked nucleocapsids from reaching the nucleus and the generation of cccDNA [71]. However, the microtubule-depolymerizing chemicals have severe side effects limiting the evidence of these results. Follow-up investigation showed that dynein light-chain LL1 works as a functional interaction partner linking capsids to the dynein motor complex, providing molecular details of HBV translocation through the cytoplasm. It is assumed that the CTD is relevant for Dynein LC binding. [74]. In addition, the relevance of actin filaments for intracellular nucleocapsid transport has been described [65]. The experimental investigation is challenging, because in the infection process the number of viral particles entering the cell is very low and the number of nucleocapsids released in the cytoplasm is even smaller.

## 3. Nuclear Transport

The life cycle of the DNA virus HBV requires the transport of the viral genome to the nucleus. The nucleocapsid is transported by a directed transport to the nuclear pore complex. The size of the capsid with 32 or 36 nm diameter is close to the upper limit for transport through the pore. It has been a matter of debate whether the capsid is imported into the nucleus or not. Using digitonin-permeabilized cells it was claimed that in contrast to immature capsids mature capsids, due to a maturation dependent exposure of a NLS, have the capacity to enter the nucleus [35,75,76]. The HBV capsid protein contains nuclear location signals (NLSs) in the C-terminus [35,77], and the NLS was shown to bind to the nuclear pore complexes (NPCs) via an interaction with nuclear transport factors (importins (karyopherins) alpha and beta) in digitonin-permeabilized mammalian cells [75]. However, cryoelectron microscopy revealed that the C-terminal part of core protein is not surface-exposed in capsids [25]. But it should be mentioned that there are reports describing that a variety of factors might affect the topology of the CTD (see below) [78]. Moreover, in permeabilized HeLa cells a specific interaction of single NPCs with capsids lacking the NLS could be demonstrated by fast wide-field fluorescence microscopy at 50 frames/s [79]. It also needs to be considered that the permeabilization procedure (digitonin) applied in these studies might affect the integrity of the cell and even the stability of the nucleocapsids. Independent from this, there are reports describing the hypothesis that rcDNA deproteinization is associated with a conformational change of the nucleocapsid, exposing the C-terminal part harboring a NLS to the surface of the nucleocapsid, thereby enabling the interaction with karyopherin α and β at the nuclear pore complex [80].

In contrast to studies based on permeabilized cells, experiments were performed based on intact cells that were incubated with membrane permeable HBV nucleocapsids harboring a Pol-linked HBV genome or reporter gene. Using this system, import of the genome into the nucleus and subsequent expression were observed. Further analyses revealed that the transport of the nucleocapsid ends at the nuclear pore complex, where dissociation of the nucleocapsid occurs, leading to the release of the Pol-conjugated HBV-genome or reporter gen (eGFP) [65]. Later studies provided further evidence that capsids interact with the cytosolic face of the NPC, where disassembly to core dimers occurs. The core dimers can translocate deeper into the nuclear basket mediated by nuclear import receptors. After dissociation of the import factors on the nuclear side of the pore, reassembly to capsids can occur [81]. Core from a patient with nuclear core stain was found to be assembled to capsids, and isopycnic ultracentrifugation of these capsids revealed that they lack nucleic acids [82]. It was described that rephosphorylation of HBc at both NTD and CTD by the packaged CDK2, following the CTD dephosphorylation that occurs during NC maturation, facilitates dissociation of the nucleocapsid [83].

The disassembly of the nucleocapsid at the nuclear pore complex raises the question about the import of the viral genome into the nucleus. As the viral DNA-genome is covalently linked to a Tyr-residue of the about 90 kDa-sized viral polymerase, a simple diffusion in the nucleus can be excluded due to the size limit of about 42 kDa for this process. A detailed analysis of the HBV polymerase identified a bipartite NLS in the TP-domain of the polymerase. The functionality of this bipartite NLS depends on casein kinase II (CKII)-dependent phosphorylation that is involved in the regulation of the genome import [84].

In the nucleus, the RC-DNA is converted to a covalently closed circular (ccc)DNA that serves as a template for subsequent transcription of viral RNAs. The viral transcripts encompass the pregenomic RNA (pgRNA, 3.5 kB) and the subgenomic RNAs (2.4, 2.1 and 0.7 kB).

## 4. Capsid Maturation

**Figure 4:**
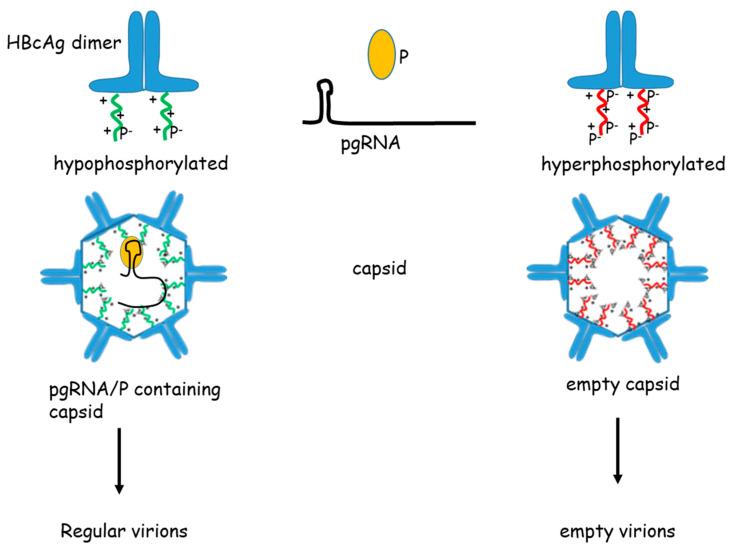
Capsid phosphorylation and pgRNA packaging. The schema summarizes a mechanism that hyperphosphorylation of the core protein impairs packaging of the pgRNA/polymerase (P) complex, while hypophosphorylation enables efficient packaging of the pgRNA/polymerase complex. (pg = pregenomic, P = HBV-polymerase)

The process of capsid maturation involves encapsidation of the pgRNA, reverse transcription of the pgRNA to generate a single stranded (-)-DNA, followed by the synthesis of the (+)-DNA. Encapsidation of the pgRNA into nucleocapsids is a selective process that depends on specific pgRNA protein interactions. The specific interaction was defined to a 137 nucleotide sequence near the N-terminus of the pgRNA, which can form a short cis-acting sequence called “ε” [85]. Part of this sequence adopts a stem-loop structure containing the bulge and the loop, which is competent for encapsidation. The bulge region was shown to be more tolerant to sequence changes than the loop [86,87]. This site has been assumed to initiate the formation of replication-competent nucleocapsids. Polymerase is preferentially encapsulated by binding primarily to pgRNA, from which it is synthesized [88]. Once the encapsidation is completed, the pgRNA is then reverse transcribed into single-stranded (-)-DNA, which is subsequently replicated to partially double-stranded DNA, giving rise to a new molecule of rcDNA. Immature nucleocapsids (early pgRNA-containing capsids or single stranded (-)-DNA) can be found within infected cells, while mature nucleocapsids with partially double-stranded DNA are observed in released viral particles [89]. This observation has led to the hypothesis that a maturation signal in the capsid can be sensed by the envelopment machinery preventing the immature capsids from budding [90]. Indeed, the difference between mature and immature capsids can be reflected in the phosphorylation/dephosphorylation status on the core protein (Figure 4) [91]. The events take place in the CTD, which is rich in arginine residues and harbors serine phosphorylation sites. Individual and multiple substitutions in the phosphorylation sites effect the RNA encapsidation [42,78,92,93]. While phosphorylation is required for efficient RNA packaging and DNA synthesis, dephosphorylation may trigger envelopment and secretion [91]. Moreover, phosphorylation of the CTD is relevant for the reduction of nonspecific RNA encapsidation [78]. However, recent reports showed that phosphorylation or dephosphorylation, though associated with capsid maturation, show little detrimental effect in secretion of empty HBV virions, which is independent of either viral RNA packaging or DNA synthesis [34]. In accordance to this, a recent study described that CTD phosphorylation does not significantly modify the structure of the capsid to generate a structural signal triggering envelopment [94]. Rather, the capsid envelopment is proposed to be regulated by a “Single Strand Blocking” model, whereby a single-stranded nucleic acid within the capsid negatively regulates capsid envelopment [95]. A hydrophobic pocket close to the spike with the nucleocapsid, which is required for the interaction of the PreS1 domain, is changed during maturation [96]. The phosphorylation and dephosphorylation process of capsid maturation requires also the viral components and cellular factors, but the mechanism is still up for discussion. For example, the viral HBx protein, a regulatory protein that activates signal transduction, could stimulate the phosphorylation of core protein [97]. Several cell kinases, such as serine/arginine-rich protein kinase 1 and 2 (SRPK1, SRPK2) [78,98], cyclin-dependent kinase 2 (CDK2) [99], PKC [100] and polo-like kinase 1 (PLK1) [101] have been described to be involved in HBV core phosphorylation. Moreover, capsid maturation is associated with changes in core structure and size, affecting the diameter of the pores in the nucleocapsid. Recent reports provided evidence that Rab33B is a crucial factor for the assembly and stability of capsids and their trafficking to budding sites. Silencing of Rab 33B decreases the membrane association of core and, thereby, favors aberrant core/capsid accumulations that are subjected to degradation. The relevance of Rab33B for the life cycle of HBV is underlined by the observation that in HBV positive cells the expression of Rab33B is increased [102].

## 5. Envelopment

Mature nucleocapsids have three alternative fates, either extracellular secretion as naked capsids, disassembly to deliver their rcDNA into the nucleus for conversion into cccDNA or release as viral particles enveloped with viral surface proteins (Figure 2). LHBs and SHBs are essential for the capsid envelopment, but MHBs are not [103]. Expression of SHBs appears to be sufficient for the formation and secretion of subviral spheres, whereas the co-expression of the SHBs and LHBs has been shown to be strictly required for the production of mature viral particles [104]. The PreS1/PreS2 domain has a dual membrane topology. The fraction of the PreS1/S2 domain of LHBs that faces the cytoplasm is involved in the capsid envelopment [11,12]. N-terminal fusion of a secretion signal sequence to LHBs that impairs the co-translational translocation, thus preventing the cytosolic orientation of a fraction of the PreS1/S2 domain, leads to invalid secretion of viral particles, which indicates the role of the cytosolic form of LHBs in the virion assembly and formation [105]. Point mutations in the PreS1 domain between arginine 103 and serine 124, the so-called matrix domain (MD), by introducing alanine residues, block virion formation and propose that C-terminal PreS1 and N-terminal PreS2 are essential for HBV envelopment [14]. Meanwhile, the N-terminal 102 aa in the PreS1 domain has been assumed for quite a long time to be dispensable for virion envelopment and secretion [106]. In contrast, peptides derived from the N-terminus of the PreS1 domain, selected from a random hexapeptide library, displayed on filamentous phage bound to full-length HBcAg [107], suggesting a role of the N-terminal part of PreS1 domain in viral envelopment. Based on a variant isolated from a patient suffering from chronic HBV that lacks aa 25–39 in the N-terminus of PreS1, it was found that the N-terminal PreS1 domain determines LHBs secretion and triggers proper assembly of HBV virions. This deletion caused a significant fraction of semi-enveloped viral particles, a variant that has not been described before [108]. The discrepancy between these studies could be based on the different experimental strategies. In the study from Jiang et al. [108], HBV particle gel essay, HBV mixed ELISA and electron microscopy were mainly used to discriminate partially-enveloped viral particles from fully assembled viral particles. The above mentioned study by Bruss et al. [106] performed immuno-precipitation and subsequent radioactive endogenous polymerase reaction (EPR) to quantify HBV viral particles, which fails to distinguish between partially-enveloped virions and intact enveloped virions.

In addition to the PreS1/S2 domain that participates in the envelopment, a cytosolic S loop between TM1 and TM2 was shown to bind efficiently to the nucleocapsid by analysis of the binding affinity between HBV core particles and various synthetic peptides [109]. The function of this region in nucleocapsid envelopment is likely to rely on a structural conformation, as an insertion within this region was tolerated [110]. Besides, some specific regions of nucleocapsid residues have been involved in nucleocapsid envelopment. A peptide derived from LHBs bound at the tips of the spikes in the capsids can reduce the production of virions [111]. Mutations clustering at a ring-like groove around the base of the spike and at a small area on the capsid surface close to the pore allowed nucleocapsid formation, but blocked particle envelopment and virion formation [17]. The direct interaction between capsid and surface proteins is suggested by a structural model based on electron microscopy analysis of HBV particles derived from patients [27]. However, a specific contact between capsid and surface protein could not be observed from another report [112]. In contrast to this, it was described that several core amino acids are crucial for direct interaction with LHBs. These are Y132 and residues L60, L95, K96 and I126. However, the relevance of Y132 is questionable, as this mutant adopts a non-canonical structure. [113]. Moreover, an alternative model was described suggesting that the nucleocapsid provides a general template for the interaction with the envelope instead of an absolute defined inflexible interaction [112].

## 6. Release of HBV Subviral Spheres

HBV is unusual among animal viruses in that the surface proteins are not only components of virion envelopes but are also incorporated into subviral particles having the shape of spheres and filaments without any genomes and nucleocapsids. Overexpression of SHBs in eukaryotic cells enables the secretion of spheres, with a diameter of about 22 nm, consisting of lipid and approximately 100 copies of the SHBs [114]. However, SHBs synthesized in yeast is not secreted [115]. The structure of yeast-derived spheres is partially different from that produced by the mammalian cells regarding glycosylation and disulfide bridges [116]. As the SHBs alone are sufficient for self-assembly, intracellular transport and secretion, many data characterizing the assembly and secretion of spheres are based on mammalian cell lines transfected with a SHBs expression construct. Extracellular spheres have a diameter of about 22 nm and are organized as octahedral particles [117]. In contrast to the recombinant octahedral spheres, the native spheres derived from patient serum are irregularly organized with spike-like features arranged in a crystalline-like pattern on the surface, as revealed by cryo-EM [118]. The major component of spheres is SHBs, but they also contain variable amounts of MHBs and very little of LHBs. It was shown that the assembly of HBsAg into spherical particles occurs within the endoplasmic reticulum (ER), and the particles accumulate and remain within the ER for most of the time before secretion [119]. In contrast, HBsAg was shown to form the dimer rapidly in the ER and then to be transported to a pre-Golgi compartment to complete the assembly process [120]. Ultrastructural analysis of cells producing only SHBs showed that transmembrane dimers initially assemble in a filamentous form, progressively increase in size and are transported to the ER-Golgi intermediate compartment (ERGIC), where they are relaxed and converted into spheres [121,122]. Then, the spheres are transported through the constitutive secretory pathway, thereby traversing the Golgi complex. Here, high mannose carbohydrates are linked to Asn-146 of SHBs [119] (Figure 2). Studies of multiple mutations of SHBs have defined amino acids that are critical for the assembly and export of spheres. Mutations in the N-terminal signal sequence did not block the insertion into the ER bilayer, but rather the further participation in assembly [104]. In addition, mutants with deletions in the N-terminal hydrophobic and hydrophilic domains of the S protein failed to release SHBs, although SHBs entered the secretory pathway and translocated at least the second hydrophilic domain across the membrane of the ER [123]. This might reflect that the deletions affect the SHBs structure and the mutant is considered as “misfolded” and, thereby, excluded from secretion [124]. All in all, SHBs appear to be more sensitive to deletions and mutations, whereas they tolerate insertions better. For instance, the N-terminal domain was shown to be permissive for the insertion of large heterologous sequences [125].

## 7. Release of HBV Viral Particles and Filaments

It has been assumed for quite a long time that subviral particles (spheres and filaments) and viral particles self-assemble in the lumen of the endoplasmic reticulum and are exported using the same route as spheres [126,127]. They were assumed to be transported into the ER-Golgi intermediate compartment (ERGIC) and released by the general secretory pathway [121,122]. Recent work suggests that the release pathway of HBV viral particles is different from spheres. The budding and egress of HBV depends on intraluminal vesicles of maturing endosomes—the MVBs and the ESCRT (endosomal sorting complex required for transport) system [128,129,130,131,132,133,134] (Figure 2). The ESCRT pathway is initially defined by the cargos required to transport membrane proteins into vesicles that bud inward into the lumen of maturing endosomes (MVBs) [135,136]. MVBs can fuse either with lysosomes to release vesicle contents or with the plasma membrane to release extracellular exosomes [137,138]. The ESCRT-MVBs pathway components consist of the ESCRT adaptors, ESCRT-I, -II, and -III complexes, together with the Vps4 ATPase and other associated proteins. The adaptors concentrate vesicle cargoes and recruit the early-acting ESCRT factors such as ESCRT-I/-II complexes, which can bind ubiquitin and recruit late-acting ESCRT-III subunits. ESCRT-III subunits then form filaments and recruit Vps4 ATPase, which together constrict membranes and mediate fission [139,140,141]. The process of assembly and budding is often inextricable. Virus budding is usually coupled tightly to virion assembly, and most viruses; therefore, use the short peptide motifs within the structural proteins to recruit the ESCRT components. As the viruses are arrested at a very late stage, these motifs are defined as “late-assembly domains”. By now, at least three distinct classes of late-assembly domains function in enveloped virus budding. YPXL late-assembly domains bind ALIX, an ESCRT-I and -III interaction partner [142,143]. P(T/S)AP late-assembly domains interact with the TSG101 subunit of ESCRT-I [144,145]. PPXY late-assembly domains bind NEDD4 family proteins [146,147]. The discovery of new viral late-assembly domains has expanded the identification of analogous motifs to cellular proteins. A recent study revealed that the release of the HBV viral particle via the ESCRT machinery is mediated by the cellular protein α-taxilin, which harbors a YXXL motive corresponding to the late domain found in Gag protein of EIAV (equine infectious anemia virus (EIAV)). In HBV replicating cells, the expression of α-taxilin is increased leading to a significantly elevated amount of α-taxilin. Silencing of α-taxilin abolishes the release of HBV virions, while the release of spheres is not affected. Further analyses revealed that α-taxilin, on the one hand, binds to the N-terminal domain of LHBs and, on the other hand, recruits via its late domain tsg101. Thus, binding of α-taxilin to LHBs overcomes the problem that LHBs lack a late domain required for the interaction with the ESCRT-system. The α-taxilin bound to LHBs provides the late domain (YAEL) and, thereby, enables the interaction with tsg101 and the MVB-dependent release [128]. Another cellular protein γ2-adaptin, involved in the formation of intracellular transport vesicles, has also been described to mediate the interaction between virions and ESCRT/MVB. γ2-adaptin interacts exclusively with the i-PreS topology of LHBs [148], and it also recognizes the HBV core protein. This is mediated by Nedd4, which interacts with γ2-adaptin after ubiquitination and binds to the PPAY motif of core protein [149,150,151]. Meanwhile, it has been reported that ESCRT-III complex-forming CHMP proteins and the Vps4 ATPases are involved in the release of HBV virions. Co-expression of dominant negative mutants of CHMP or Vps4 ATPases significantly blocks the budding of virions [132,133,134].

Years ago, analysis of the sera from patients suffering from chronic HBV infection revealed the existence of two populations of HBV viral particles characterized by different buoyant densities. Further analysis revealed that the particles with the lower density lack endogenous polymerase activity. Electron microscopy analysis of these particles suggested that these particles are “empty” [152,153,154].

Formation of “empty” virions, lacking any DNA genome, requires the presence of empty nucleocapsids. In contrast to *Escherichia coli* (*E. coli)*-derived capsids, HBV capsids derived from liver cells lack unspecifically packaged RNA. It is assumed that, in contrast to “regular” immature nucleocapsids, these empty capsids lack signals that prevent envelopment and thus can be enveloped by the surface proteins [155].

Filaments like viral particles are characterized by a significantly higher content of LHBs than spheres. Filaments were estimated to contain LHBs, MHBs and SHBs in the ratio of 1:1:4 [156]. The cryo-EM study of the tubular particles showed that the three proteins assemble as a mixed population of homo- and heterodimers, with spike-like features projecting from the membrane [6]. Deletion of aa 25–39 in the PreS1 domain of LHBs has been shown to be associated with a decreased assembly capacity, as reflected by the reduced length of filaments and appearance of incompletely enveloped viral particles [108]. Unlike SHBs, selective overexpression of the LHBs leads to the accumulation of LHBs and the inefficient secretion of HBsAg in the supernatant. Moreover, co-expression of LHBs with either MHBs or SHBs blocked the release of HBsAg as well [157]. Co-injection of the mRNA of SHBs with LHBs appeared to favor the secretion of trace quantities, if at all, of LHBs [158]. The shape and the efficiency of the release of filaments depend on the relative amounts of SHBs and LHBs. A low level of LHBs results in the production and secretion of spheres containing LHBs, while a higher level of LHBs leads to the formation of filaments [157]. The low secretion efficiency of LHBs could be due to specific retention motifs in this protein [159] and/or the shape of the filamentous particles [160]. The accumulation of LHBs can contribute to the formation of ground glass hepatocytes, hepatocellular injury and even hepatocellular carcinoma [161,162]. Ultrathin section of filament-producing cells showed that filaments were retained in the periphery of extremely large intracellular cisternae, probably related to a pre-Golgi compartment. However, it has been reported that filamentous particles produced by transfected cells were accumulated in dilated convoluted probably ER-related compartments of cells expressing LHBs only [122]. Storage of LHBs leading to a storage disease analogous situation could be observed in hepatocytes of LHBs-transgenic mice as well [161]. But it is very interesting that in HBV-infected cells and patients filaments are frequently observed in the supernatant and serum [163,164,165]. This raises the question whether overexpression of LHBs or co-expression of LHBs with either SHBs or MHBs could mimic the authentic release pathway of HBV filaments. The MVB-dependent release of virions depends on LHBs that binds to α-taxilin and thereby enables the interaction with the ESCRT-machinery. In light of the fact that filaments differ with respect to the amount of LHBs strongly from spheres, it was analyzed if filaments are MVB-dependently released like virions due to the LHBs-dependent interaction with α-taxilin. To avoid any interference of LHBs (filament)-specific signals with signals that represent virions, a core-deficient HBV mutant was used for these studies to exclude the formation of virions. This study revealed that filaments like virions enter into MVBs, and interruption of the MVBs biogenesis or silencing of α-taxilin inhibits the release of filaments. Thus, filaments like infectious viral particles are released via the ESCRT/MVB pathway, while spheres are released via the secretory pathway, as shown in Figure 2 [129].

## 8. Release of Naked Capsids

Though it has been reported that non-enveloped (nucleo)capsids (naked capsids) are hardly detected in the bloodstream of infected patients or chimpanzees [166], naked capsids can be secreted by HBV-replicating cell lines [133,167,168]. Moreover, all patients who have been exposed to HBV can elicit high titers of anti-capsid antibodies. The nucleocapsid has a high immunogenicity. The nucleocapsid functions as a B-cell-dependent antigen that directly binds and activates B-cells to produce antibodies [169,170]. This supports the hypothesis that the nucleocapsid is released into the blood but might rapidly be cleaned by unknown reasons. How the naked nucleocapsids are released by cells is still under investigation. In contrast to HBV viral particles, the budding of naked capsids was described to be independent from the ESCRT-machinery [133,167]. In contrast, HGS (HRS, hepatocyte growth factor-regulated tyrosine kinase substrate), a component that belongs to the ESCRT-0 complex, was shown to enhance the release of naked capsids. The function of HGS on the secretion of naked capsids is related to Alix [130]. Alix exerts different roles with respect to the release of the nucleocapsid. Silencing of Alix significantly increases the budding of naked nucleocapsids and inhibits the release of virions [133]. But another study showed that ectopic overexpression of Alix enhanced capsid egress, while its depletion inhibited capsid release. Further analysis showed that the Bro1 domain of Alix binds to core and mediates capsid release [167]. A recent publication provided evidence that autophagosome-associated Rab33B, Atg5, Atg12 and Atg16L1 are involved in capsid assembly and egress, suggesting a link between capsid formation and autophagy [171].

With respect to their content, naked capsids are heterogeneous. The formation of naked capsids harboring pgRNA in vitro has been described [155]. Moreover, it was found that extracellular HBV RNAs were preferentially incorporated in naked capsids rather than in complete virions. The RNA population detected in naked capsids is heterogeneous with respect to the length of the RNA molecules. Detection of RNA-containing naked capsids is not restricted to in vitro systems. Analysis of sera from HBV patients revealed the presence of RNA-containing naked capsids [172].

## 9. Conclusions

In contrast to many other viruses, the intracellular trafficking of HBV is rather complex as apart from the infectious viral particle, a variety of additional subviral particles is formed, transported and finally released by HBV-replicating cells. Various HBV-specific particles are released by different pathways. In the last years, interference with the nucleocapsid or virus morphogenesis and intracellular trafficking came more into the focus as a potential antiviral strategy. It is obvious that inhibition of virus release could represent an efficient tool to prevent the spread of viral infection. Moreover, intracellular retention can lead to an increased degradation of viral structural protein and subsequent MHC-class I-dependent presentation of HBV-specific peptides on the cell surface that enables the recognition and subsequent destruction by CTLs. However, intracellular retention of viral particles could lead to an elevated intracellular level of HBV genomes that could lead to an elevated frequency of integration events of viral DNA into the host genome. A more detailed understanding of the intracellular trafficking of HBV could enable the detour of intracellular genomes directly to degradation systems (i.e., the lysosomal compartment) and, thereby, improve the benefit:risk ratio of these approaches.
